# GSEApy: a comprehensive package for performing gene set enrichment analysis in Python

**DOI:** 10.1093/bioinformatics/btac757

**Published:** 2022-11-25

**Authors:** Zhuoqing Fang, Xinyuan Liu, Gary Peltz

**Affiliations:** Department of Anesthesia, Pain and Perioperative Medicine, Stanford University School of Medicine, Stanford, CA 94305, USA; Department of Otolaryngology-Head and Neck Surgery, Stanford University School of Medicine, Stanford, CA 94305, USA; Department of Anesthesia, Pain and Perioperative Medicine, Stanford University School of Medicine, Stanford, CA 94305, USA

## Abstract

**Motivation:**

Gene set enrichment analysis (GSEA) is a commonly used algorithm for characterizing gene expression changes. However, the currently available tools used to perform GSEA have a limited ability to analyze large datasets, which is particularly problematic for the analysis of single-cell data. To overcome this limitation, we developed a GSEA package in Python (GSEApy), which could efficiently analyze large single-cell datasets.

**Results:**

We present a package (GSEApy) that performs GSEA in either the command line or Python environment. GSEApy uses a Rust implementation to enable it to calculate the same enrichment statistic as GSEA for a collection of pathways. The Rust implementation of GSEApy is 3-fold faster than the Numpy version of GSEApy (v0.10.8) and uses >4-fold less memory. GSEApy also provides an interface between Python and Enrichr web services, as well as for BioMart. The Enrichr application programming interface enables GSEApy to perform over-representation analysis for an input gene list. Furthermore, GSEApy consists of several tools, each designed to facilitate a particular type of enrichment analysis.

**Availability and implementation:**

The new GSEApy with Rust extension is deposited in PyPI: https://pypi.org/project/gseapy/. The GSEApy source code is freely available at https://github.com/zqfang/GSEApy. Also, the documentation website is available at https://gseapy.rtfd.io/.

**Supplementary information:**

Supplementary data are available at *Bioinformatics* online.

## 1 Introduction

Gene set enrichment analysis (GSEA) (Subramanian *et al.*, 2005) is a commonly used method for assessing whether a gene expression pattern measured in a test set of cells or tissues is similar to one previously found in other cell types, disease conditions or treatment responses. For example, GSEA has been used to identify genes/pathways associated with treatment response or disease prognosis (Verstockt *et al.*, 2019; Wang *et al.*, 2019; Labrecque *et al.*, 2019), and to identify stem cell signatures in human cancer tissues (Merlos-Suarez *et al.*, 2011; Corominas-Faja *et al.*, 2013). We previously used GSEA to demonstrate that the collagen-producing myofibroblasts in mutated hepatic organoids were similar to those in fibrotic liver tissue obtained from patients with liver cancer or commonly occurring fibrotic liver diseases (Guan *et al.*, 2021). GSEA calculates a normalized enrichment score (NES), which indicates how similar the differentially expressed gene sets are between the test and comparator datasets. However, single-cell RNA-sequencing (scRNA-Seq) is now commonly used for transcriptomic characterization of cells, organoids and tissues. When the datasets generated from scRNA-Seq are huge and provide large amounts of information, the computational challenges associated with them increase (Kiselev *et al.*, 2019; Lakkis *et al.*, 2019). Although there have been several different implementations can be used for GSEA analysis, such as GSEA-R (Subramanian *et al.*, 2005), GSEA-P (Subramanian *et al.*, 2007), fGSEA (Korotkevich *et al.*, 2021), only GSEApy (released in early 2017) and a recently published tool, named blitzGSEA (Lachmann *et al.*, 2022), are available for Python computing environment. Furthermore, GSEApy ships with additional features that facilitate enrichment analysis, including an application programming interface (API) to Enrichr web service (Chen *et al.*, 2013; Kuleshov *et al.*, 2016; Xie *et al.*, 2021), an API to BioMart web service (Durinck *et al.*, 2005), the single sample GSEA methodology (Barbie *et al.*, 2009) and a utility for gene set over-representation analysis. GSEApy was implemented by Numpy with process-based parallel computing support initially. However, this implementation is not well optimized in speed and has limited utility for analyzing larger scRNA-seq datasets that require memory of more than 32 GB (Lachmann *et al.*, 2022).

To enable enrichment analysis to be performed on large-scale data, we re-implemented GSEApy in a high-performance programming language (Rust). It provides better memory security, comparable speed to that of C/C++ and the same API as the previous version (≤ v0.10.8). Furthermore, the Rust extension of GSEApy can take full advantage of modern computers' multi-threading parallel computing capabilities because Python’s global interpreter lock was released. When small gene set libraries are analyzed (e.g. 278 pathways in this article), it runs 3-fold faster but with four times less memory usage (Supplementary Fig. S1A). For large gene set libraries (e.g. 2860 pathways in this article), it can run 80-fold faster without much more memory (Supplementary Fig. S1B).

## 2 Implementation

The GSEApy internal is written in Rust, and it consists of six tools:

- **gsea**: Perform enrichment analysis using the GSEA statistical methodology.
- **prerank**: Pre-ranked GSEA, particularly for datasets with a small number of replicates.
- **ssgsea**: Single sample GSEA (ssGSEA) methodology.
- **replot**: Re-generate enrichment plots from the GSEA-P output files.
- **enrichr**: Perform an over-representation analysis on a list of genes. It supports several organisms, including human, mouse, fly, yeast, zebrafish and *C.elegans*.
- **biomart**: Convert gene ids with the BioMart API.

The GSEApy will automatically obtain gene set libraries from the Enrichr web service for analysis. To facilitate the interpretation of enrichment results, GSEApy provides several visualization methods (gseaplot, heatmap, dotplot, barplot and ringplot). We also provide a complete documentation website, including a user guide describing all of these tools, examples and frequently asked questions.

## 3 Results

### 3.1 Computational efficiency improvement by Rust

The most computationally expensive part of GSEA is generating the null distribution of Enrichment Scores (ES) for the *P*-value calculation. GSEA performs random permutations of either the samples or the gene labels to obtain the null distribution. One thousand permutations are set as the default parameter, which produces 1000 ES. An observed ES is then compared with the 1000 shuffled ES to calculate a *P*-value. We previously implemented a Numpy version that performed the GSEA. However, the Numpy version consumed a huge amount of memory while using multi-CPUs, an issue reported by many GSEApy users. To address this, we re-implemented the GSEA algorithm in Rust. When we compared the time and memory cost between the Numpy (v0.10.8) and Rust version with a gene permutation experiment (22 922 genes, 278 or 2860 pathways, 1000 permutations), we found that the Rust implementation was 3- to 80-fold faster in run time with one thread (Supplementary Fig. S1A). The run time with eight threads was decreased to a few minutes for both versions, but the Rust version was still 2-fold faster, and the memory cost was reduced from 50 GB to 1.4 GB (for the 2860 pathways) (Supplementary Fig. S1B). We also compared the execution speed of fGSEA, blitzGSEA, GSEA-P, GSEA-R and GSEApy on a single thread (Supplementary Fig. S1C). While it took over 1 hour for GSEA-R to calculate the statistics for 2860 pathways, GSEA-P and GSEApy took 3.7 and 5.1 minutes, respectively. fGSEA and blitzGSEA outperformed the other tools since their execution time was <1 minute. In a sample permutation experiment, the Rust binding version was almost 3-fold faster than GSEA-R (Supplementary Fig. S1D), and the Numpy and Rust versions had similar memory costs for small datasets (Supplementary Fig. S1E).

### 3.2 Enrichment analysis and data visualization

**Fig. 1.**
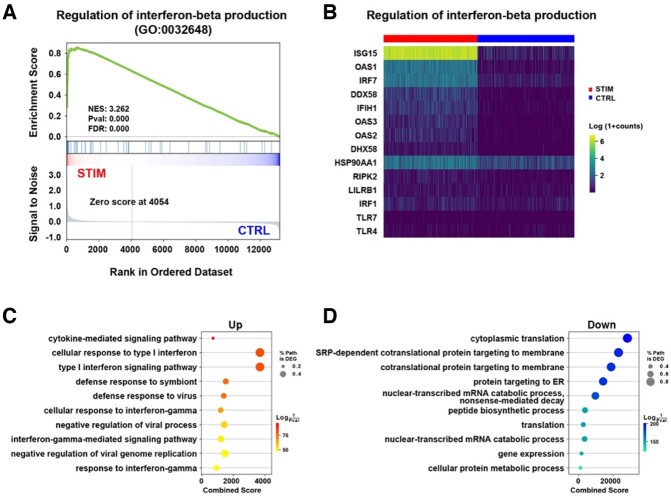
An example of enrichment analysis performed using GSEApy. (**A**) GSEA enrichment plot of the regulation of interferon-beta production pathway. (**B**) A heatmap shows the leading-edge genes that appear in the ranked list at or before the point at which the running ES reaches its maximum deviation from zero identified in (A). (**C**, **D**) over-representation analysis shows the top 10 significantly enriched pathways of up-regulated genes (C) and down-regulated genes (D), respectively. % Path is DEGs, the percentage of DEGs that overlapped with the pathway of interest. Combined score is defined by the Enrichr (Xie *et al.*, 2021)

The improvement in computational efficiency enables GSEApy to perform enrichment analysis for large-scale samples. To illustrate how GSEApy could be used for biological discovery, we analyzed a publicly available scRNA-seq dataset (GSE96583) of peripheral blood mononuclear cells (PBMCs) obtained from control and interferon β-treated subjects (Kang *et al.*, 2018). After generating the log normalized counts using Scanpy (Wolf *et al.*, 2018), we analyzed the annotated subset of CD14^+^ monocytes (2215 control and 2147 stimulated cells). We performed GSEA (1000 permutations, sample permutation and 6036 pathways) on the 4362 transcriptomes of the CD14^+^ monocytes. The interferon β production pathway was significantly enriched (*P*-value < 0.01, False Discovery Rate (FDR) < 0.01, NES = 3.262) in the stimulated CD14^+^ monocytes (Fig. 1A and B). Next, we identified 1717 differentially expressed genes (DEGs) (Wilcoxon test with FDR < 0.05) using the rank_genes_groups function in Scanpy. An over-representation analysis (GSEApy’s Enrichr API) was performed on the 687 up-regulated and the 1030 down-regulated DEGs. The up-regulated genes indicated that the interferon signature was activated in the interferon β-stimulated cells (Fig. 1C), while the down-regulated genes were associated with general cellular functions, such as protein translation (Fig. 1D). This analysis confirms the effect of interferon treatment, which indicates that this dataset can be used for subsequent investigations.

## 4 Conclusion

GSEApy provides a fast and straightforward way to perform enrichment analysis. It requires minimal arguments, provides clear output and operates from both the command line and Python environment, which maximizes the ease of use, accommodates novice programmers and supports large-scale dataset analysis. It also can quickly generate high-quality, publication-ready plots. GSEApy will be one of the fundamental packages for enrichment analysis in Python.

## Supplementary Material

btac757_Supplementary_Data

## Data Availability

The GSEApy source code is freely available at https://github.com/zqfang/GSEApy.
