# Supplementary material for: GSEApy: a comprehensive package for performing gene set enrichment analysis in Python
Source: Bioinformatics. 2022 Nov 25;39(1):btac757. doi: 10.1093/bioinformatics/btac757 (PMC9805564; doi:10.1093/bioinformatics/btac757)
Supplement: btac757_Supplementary_Data [file btac757_supplementary_data.docx]

**Supplemental Information**

**GSEApy: a comprehensive package for performing gene set enrichment analysis in Python**

Zhuoqing Fang^1^, Xinyuan Liu^2^ and Gary Peltz^1*^

^1^ Department of Anesthesia, Pain and Perioperative Medicine. Stanford University School of Medicine, Stanford CA 94305

^2^ Department of Otolaryngology-Head and Neck Surgery, Stanford University School of Medicine, Stanford, CA, United States

**Contact**: [gpeltz@stanford.edu](mailto:gpeltz@stanford.edu)

**Figure S1**


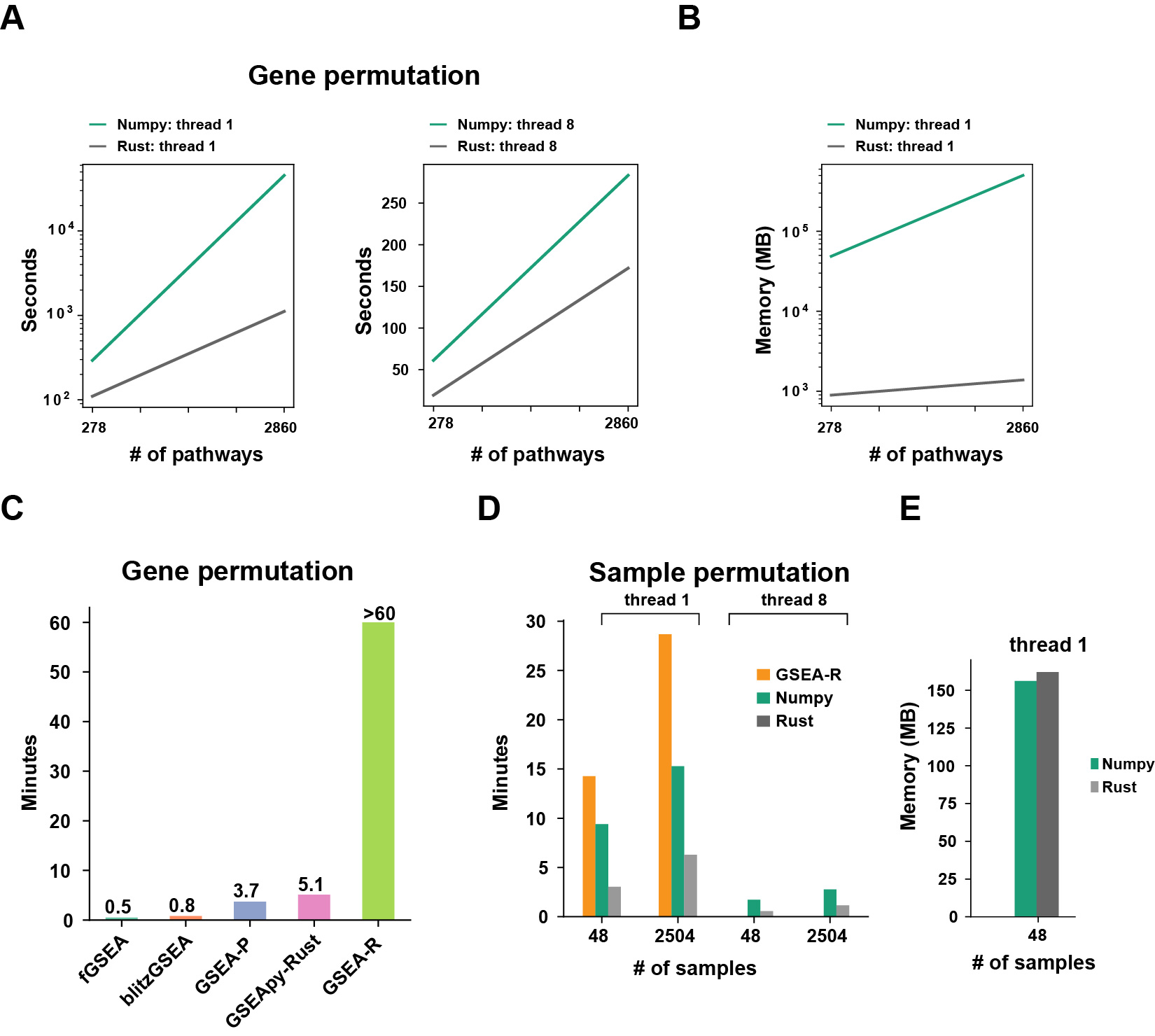


**Figure S1**. **Computational efficiency comparison between GSEApy (v0.10.8) and Rust implementation (v0.13.0)**. (**A)** Running time usage and (**B**) memory cost of a gene label permutation experiment (Prerank tool). A pre-ranked gene list (with 22922 genes) was used to conduct enrichment analysis with the following parameters: 1000 permutations, 278 or 2860 pathways. Experiments were tested on 1 or 8 threads (CPUs). (**C**) Comparison of the execution speed for fGSEA (Korotkevich, et al., 2021), blitzGSEA (Lachmann, et al., 2022), GSEA-P (Subramanian, et al., 2007), GSEApy, GSEA-R (Subramanian, et al., 2005) on single thread. A gene label permutation experiment (Prerank tool) was run with parameters: 2680 pathways, 1000 permutations, and 22922 genes. The running time of GSEA-R exceeded 60 minutes. (**D**) Running time usage of a sample label permutation experiment (GESA tool) with two sets of parameters: 48 samples, 9020 genes, 2680 pathways, 1000 permutations; 2504 samples, 12273 genes, 2084 pathways, 1000 permutations. Multi-threading is not supported in GSEA-R (v1.2). (**E**) Memory cost of a sample label permutation experiment with parameters: 48 samples, 9020 genes, 2084 pathways, 1000 permutations.

**References**

Korotkevich, G.*, et al.* Fast gene set enrichment analysis. *bioRxiv* 2021:060012.

Lachmann, A., Xie, Z. and Ma'ayan, A. blitzGSEA: Efficient computation of Gene Set Enrichment Analysis through Gamma distribution approximation. *Bioinformatics* 2022.

Subramanian, A.*, et al.* GSEA-P: a desktop application for Gene Set Enrichment Analysis. *Bioinformatics* 2007;23(23):3251-3253.

Subramanian, A.*, et al.* Gene set enrichment analysis: a knowledge-based approach for interpreting genome-wide expression profiles. *Proceedings of the National Academy of Sciences of the United States of America* 2005;102(43):15545-15550.
